# The Most Common Location of Schools with Viral Upper Respiratory Tract Infection Clusters in Taiwan, 2011–2019

**DOI:** 10.3390/children9050720

**Published:** 2022-05-13

**Authors:** Fu-Huang Lin, Yu-Ching Chou, Wu-Chien Chien, Chi-Hsiang Chung, Chi-Jeng Hsieh, Chia-Peng Yu

**Affiliations:** 1 School of Public Health, National Defense Medical Center, Taipei City 11490, Taiwan; noldling@ms10.hinet.net (F.-H.L.); trishow@mail.ndmctsgh.edu.tw (Y.-C.C.); chienwu@ndmctsgh.edu.tw (W.-C.C.); g694810042@gmail.com (C.-H.C.); 2 Department of Medical Research, Tri-Service General Hospital, National Defense Medical Center, Taipei City 11490, Taiwan; 3 Graduate Institute of Life Sciences, National Defense Medical Center, Taipei City 11490, Taiwan; 4 Taiwanese Injury Prevention and Safety Promotion Association, Taipei City 11490, Taiwan; 5 Department of Health Care Administration, Asia Eastern University of Science and Technology, New Taipei City 22061, Taiwan; fl004@mail.aeust.edu.tw

**Keywords:** influenza, respiratory syncytial virus, cluster, epidemiology, upper respiratory tract infection, retrospective study

## Abstract

Clusters of acute upper respiratory tract infections are mainly caused by type A or B influenza virus. Numerous factors modify the risk of upper respiratory tract infection (URTI) cluster transmission. The purpose of this study was to investigate the epidemiological characteristics, differences, and epidemic trends in influenza viruses and in non-influenza respiratory pathogens, and the distribution of the sites of URTI cluster events in Taiwan from 2011 to 2019. We examined the publicly available annual summary data on 1864 confirmed URTI clusters in the Taiwan Centers for Disease Control (Taiwan CDC) from 2011 to 2019. URTI clusters were mainly divided into 1295 clusters of influenza virus infections, 149 clusters of non-influenza respiratory pathogen infections, 341 clusters of pathogens not detected by routine tests, and 79 clusters of unchecked samples. There were statistically significant differences (*p* < 0.001) in the event numbers of URTI clusters among influenza and non-influenza respiratory pathogens between 2011 and 2019. There were statistically significant differences (*p* = 0.01) in instances of URTI clusters among non-influenza respiratory pathogens between 2011 and 2019. There were also statistically significant differences (*p* < 0.001) in instances of URTI clusters in different locations between 2011 and 2019. In all the pathogens of URTI clusters (odds ratio (OR) = 1.89–2.25, *p* = 0.002–0.004), most single infections were influenza A viruses (64.9%, 937/1444). Respiratory syncytial virus single infections were most numerous (43.0%, 64/149) among the non-influenza respiratory pathogens of URTI clusters. Of the institutions where URTI clusters occurred, schools had the most cases (50.1%, 933/1864) (OR = 1.41–3.02, *p* < 0.001–0.04). After the categorization of isolated virus strains by gene sequencing, it was found that, of the seasonal influenza A viruses, the H1N1 subtype viruses were predominantly A/California/07/2009, A/Michigan/45/2015, and A/Brisbane/02/2018, and the H3N2 subtype viruses were predominantly A/Hong Kong/4801/2014, A/Singapore/INFIMH-16–0019/2016, and A/Switzerland/8060/2017, during 2017–2019. Of the influenza B viruses, B/Brisbane/60/2008 (B/Vic) was the dominant type, and some were B/Massachusetts/02/2012 (B/Yam) and B/PHUKET/3073/2013 (B/Yam). This study is the first report of confirmed events of URTI clusters from surveillance data provided by the Taiwan CDC (2011–2019). This study highlights the importance of long-term, geographically extended studies, particularly for highly fluctuating pathogens, for understanding the implications of the transmission of URTI clusters in Taiwanese populations. Knowledge gaps and important data have been identified to inform future surveillance and research efforts in Taiwan.

## 1. Introduction

Influenza is an acute viral respiratory disease. It is characterized by rapid outbreak, is widespread, and has serious complications. For children, the flu is more dangerous than the common cold. Children under the age of 5 years, especially those under the age of 2 years, are at higher risk for serious flu-related complications. Globally, the incidence of influenza in children under the age of 5 years is estimated to be ninety million cases per year [1]. The CDC estimates that from the 2010–2011 season to the 2019–2020 season, between 7000 and 26,000 children under the age of 5 years were hospitalized with influenza in the United States [2]. The hospitalization rate for the elderly and high-risk groups during an influenza epidemic is 2 to 4 times than that of a non-epidemic period. The hospitalization rate due to influenza infection for children younger than 5 years old is about 81 per 100,000 people, and for people older than 65 it is about 853 per 100,000 people [3].

The main pathogens causing influenza are influenza viruses. In terms of virus taxonomy, influenza viruses belong to the Orthomyxoviridae family. Depending on the antigenicity of the nucleoprotein, they can be divided into four types: A, B, C, and D, with a segmented single-stranded RNA gene body [3,4,5].

The influenza incubation period is about 1 to 4 days [3,4]. The infection is mainly caused by the inhalation of the influenza virus or contact with the secretions of patients. This disease can occur in all age groups. Its clinical symptoms mainly include fever, cough, headache, muscle pain, fatigue, runny nose, sore throat, and other symptoms, and disease severity is related to the immunity, underlying disease status, age, etc., of the patient [3,4,5]. In addition, a small number of infected people have gastrointestinal symptoms such as nausea, vomiting, and diarrhea, accompanied by respiratory symptoms [6]. Most healthy adults usually recover in 1 week, but some people have symptoms of coughing and general malaise that can last for more than 2 weeks [3,5]. High-risk groups are more likely to contract influenza and have serious complications due to their own immunity than non-high-risk groups, including the elderly, young children, pregnant women, and persons with immune insufficiency, as well as those with cardiovascular diseases, chronic lung diseases, kidneys diseases, diabetes, and obesity. These complications include bacterial pneumonia, viral pneumonia, encephalitis or encephalopathy, myocarditis or pericarditis, and Reye syndrome [7,8,9,10,11]. High-transmission groups may infect high-risk groups or people in places where transmission is likely to occur due to work factors, including medical staff in medical institutions, staff in chronic care institutions, and school students. Although it is not easy to distinguish influenza from other acute respiratory diseases in clinical diagnosis, such as common cold, laryngitis, bronchitis, or viral pneumonia, in general, the symptoms of influenza are more severe than those of common colds, the duration of the disease is longer, and other epidemiological characteristics are different.

Upper respiratory tract infection (URTI) cluster incidents are a public health issue to which global health units attach great importance [12]. Taiwan is in the northern hemisphere and is a tropical/subtropical region. Influenza viruses can be isolated throughout the year. Sporadic or cluster cases sometimes occur, and large-scale epidemics occur in autumn and winter. The Public Health Service of the Centers for Disease Control of Taiwan’s Ministry of Health and Welfare has begun to publish a summary of URTI cluster epidemics [13]. Thanks to the collection of these data, the collection and reporting of URTI clusters has significantly improved in terms of type, quantity, completeness, timeliness, and comprehensiveness. The purposes of this study were to test the empirical data of the Taiwan Infectious Disease Surveillance Reporting System over the past nine years (2011–2019), provide a retrospective historical perspective of URTI clusters, and explore the causes and trends of epidemiological changes that cause cluster infections.

## 2. Materials and Methods

### 2.1. Ethical Policy

This study does not require ethical approval as it involves information freely available in the public domain, and the analysis of open dataset sources, where the data are properly anonymized [14,15,16].

### 2.2. Definition of Confirmed Cases

1. The symptoms of URTI: they must meet one or more of the following symptoms: fever, cough, sore throat, shortness of breath, runny nose.

2. URTI cluster case definition: a case with upper respiratory tract symptoms that is related to a person, time, and place and is judged to be a suspected cluster infection that is likely to spread.

### 2.3. Data Source

This study used the Taiwan National Infectious Disease Statistics System (TNIDSS) public database and the statistics of communicable disease and surveillance reports of the Taiwan Disease Control Agency website [17]. The TNIDSS public database includes all communicable diseases in 5 categories, as stipulated by the Communicable Disease Control Act [18]. To ensure information security and prevent the leakage of case privacy, the system’s database does not store any personal information such as the medical history of patients, or signs and symptoms, only secondary data with statistical values. This study used processed data sets without any personal identifiers and did not use any of the data sets in such a way that individuals might be identified. The Taiwan CDC database includes information on trends, the number of upper respiratory tract clusters, and the number of respiratory viruses isolated in the country. The data on disease are collected by integrating various nationwide disease surveillance systems. These include the Real-Time Outbreak and Disease Surveillance System, the Notifiable Disease Surveillance System, The School-Based Surveillance System, the Symptom Surveillance and Reporting System, the Infectious Disease Surveillance System for Institutions with Dense Populations, and the Preventive Inoculation Information System. All of the above are accessible and linked to one another via the internet so that everyone can use the relevant outbreak information to prevent infectious disease [19].

In addition, this study uses data from the “Viral Infectious Disease Contract Laboratory Surveillance System”. The specimens mainly come from suspected patients in the outpatient, emergency, and inpatient clinics of the medical center where the contract laboratory is located, as well as suspected patients in about 165 scheduled medical examination points nationwide. Patients with suspected influenza must meet the definition of influenza-like cases (fever above 38 °C and respiratory symptoms, with one of the symptoms being muscle soreness, headache, and extreme burnout, excluding mild rhinitis, tonsillitis, and bronchitis). In this study, the genetic sequence data of the influenza virus were analyzed for each year.

### 2.4. Data Analysis

The research structure was as follows: the retrospective surveillance database analysis of all local URTI cluster events in the database from 2011 to 2019. We determined the number of URTI clustering events from 2011 to 2019 and examined the differences and trends in the distribution of their epidemiological characteristics (types of URTIs causing pathogens and URTI clustering sites). We used the numbers (N) and percentages (%) of categorical variables to present pathogen detections and pathogen infections (including influenza viruses, bacteria, and other pathogens) in each year for URTI clusters. Chi-squared tests or Fisher’s exact tests (when expected numbers in cells were less than 5) were used to examine the differences in the distributions of the categorical variables. To estimate the effect of different types of pathogens (e.g., single influenza A virus vs. non-single influenza A virus, respiratory syncytial virus vs. non-respiratory syncytial virus) and different institutions (e.g., school vs. non-school) on the risk during different calendar years (e.g., in 2011 vs. other years (combined numbers of other years)), the logistical model was the estimated odds ratio with 95% CIs. All statistical analyses were performed using SPSS software (IBM SPSS Statistics 21; Asia Analytics Taiwan, Taipei, Taiwan). All statistical tests were 2-sided with an alpha level of 0.05. The *p*-values of less than 0.05 were considered statistically significant.

## 3. Results

In this study, the number of cluster cases of respiratory infections recorded in the communicable disease and surveillance report published by the Taiwan CDC from 2011 to 2019 were analyzed. There were a total of 1864 cases (76, 72, 118, 154, 178, 166, 358, 255, and 487 cases per year in order of year, ascending) (Table 1). The number of URTI clusters caused by pathogens of respiratory influenza viruses from 2011 to 2019, (43, 46, 65, 89, 142, 90, 282, 168, and 370 per year in ascending order) and a total of 1295 cases were analyzed (Table 1). There were statistically significant differences (*p* < 0.001) in numbers of URTI clusters among respiratory pathogens between 2011 and 2019 (Table 2). Furthermore, the number of URTI clusters of non-influenza pathogens from 2011 to 2019 were studied. The aggregated data were 14, 5, 15, 13, 8, 20, 28, 31, and 15 per year in ascending order, and 149 in total (Table 3). There were statistically significant differences (*p* = 0.01) in numbers of URTI clusters among non-influenza pathogens between 2011 and 2019 (Table 3). The occurrence of URTI clusters in various institutions between 2011 and 2019 was also studied. The aggregated data were 76, 72, 118, 154, 178, 166, 358, 255, and 487 per year in ascending order, with 1864 cases in total (Table 4). There were also statistically significant differences (*p* < 0.001) in the numbers of URTI clusters in different public places between 2011 and 2019 (Table 4).

After the categorization of isolated virus strains by gene sequencing, it was found that of the seasonal influenza A viruses, the H1N1 subtype viruses were predominantly A/Brisbane/02/2018. The H3N2 subtypes were predominantly A/Singapore/INFIMH-16–0019/2016, while the rest were A/Switzerland/8060/2017 and A/Hong Kong/4801/2014. Of influenza B viruses, B/Colorado/06/2017(B/Vic) was the dominant type, and some were B/Washington/02/2019(B/Vic) and B/PHUKET/3073/2013(B/Yam) in Taiwan in 2019 (Table 5).

**Figure 1:**
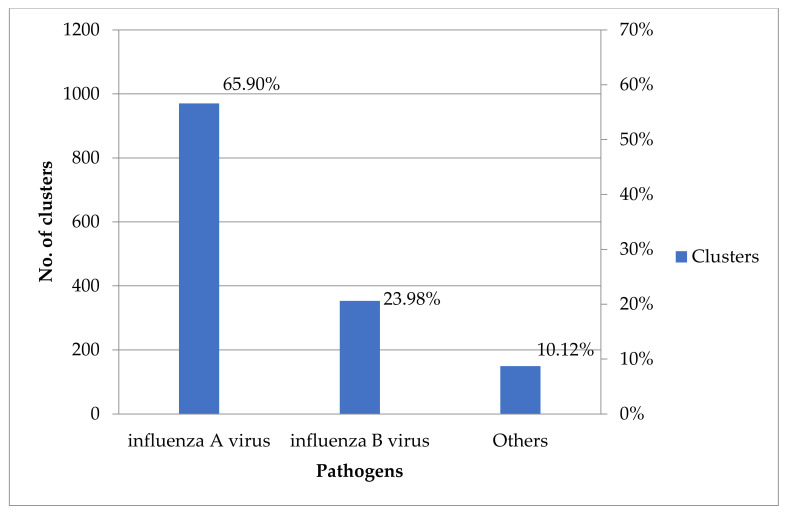
The number and percentage of influenza viruses and other pathogens among URTI clusters, Taiwan, 2011–2019. URTI: upper respiratory tract infection.

**Figure 2:**
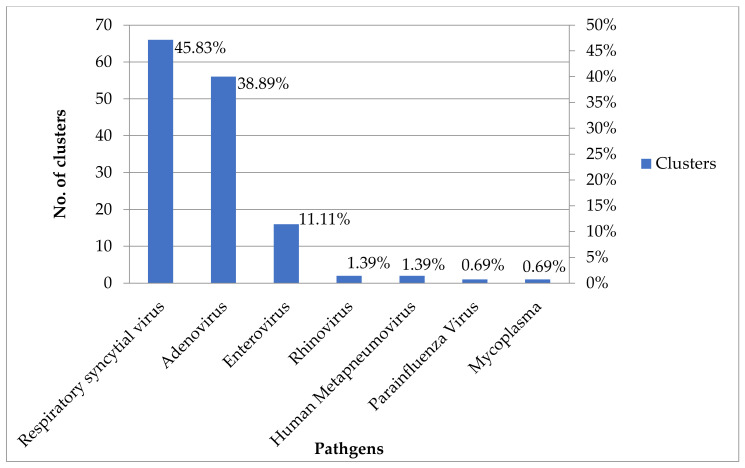
The number and percentage of non-influenza respiratory viruses among URTI clusters, Taiwan, 2011–2019. URTI: upper respiratory tract infection.

**Figure 3:**
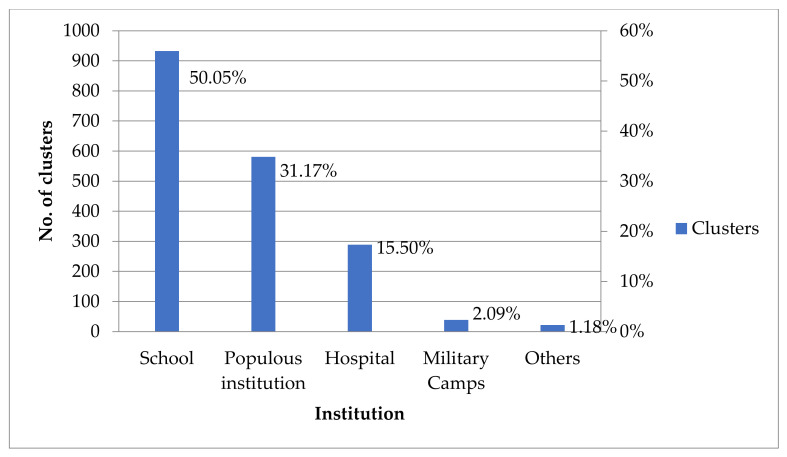
The number and percentage of institutions among URTI clusters, Taiwan, 2011–2019. URTI: upper respiratory tract infection.

Influenza A virus infections were most common (65.90%, 970/1472) in URTI clusters, and influenza B virus infections were the second most common (23.98%, 353/1472) between 2011 and 2019 (Figure 1). Among non-influenza viruses, respiratory syncytial virus infections were the most common (45.83%, 66/144) in URTI clusters, and adenovirus infections were the second most common (38.89%, 56/144) in URTI clusters between 2011 and 2019 (Figure 2). Schools were the most common institutional location (50.05%, 933/1864) of URTI clusters, and “populous institutions” were the second most common (31.17%, 581/1864) location of URTI clusters between 2011 and 2019 (Figure 3).

## 4. Discussion

Schools were the most common location of viral URTI clusters in Taiwan, 2011–2019. Influenza A virus was the main cause of the viral URTI clusters. The respiratory syncytial virus was the main cause of the non-influenza URTI clusters. This study confirmed that the combination of locations at high risk of URTI clusters (schools, populous institutions, and hospitals) and etiological substances (influenza A virus, influenza B virus, and respiratory syncytial virus) may lead to URTI clusters. In addition, the event numbers of URTI clusters showed an increasing trend during 2015–2017 in schools, both for pathogenic virus (influenza A virus) and for non-influenza respiratory virus (adenovirus).

Influenza A virus is the main pathogen responsible for URTI cluster incidents in Taiwan. It is the main pathogen, accounting for approximately 65.9% of all known pathogens (970/1472). It can also be seen that the number of influenza clustering incidents has been increasing year-by-year (43 cases in 2011 rose to 370 cases in 2019), which means that influenza clusters are still a gap in Taiwan’s epidemic prevention. It is recommended that the government should form an expert committee to propose effective epidemic-prevention strategies and should jointly fight epidemics together with the public to prevent or slow down their spread. In addition, some studies have reported that influenza outbreaks are seasonal, causing large-scale illnesses and severe disease transmission [20].

Influenza viruses in the upper respiratory tract are transmitted through droplets and contact and thus are prone to rapid spread in crowded places and often cause large-scale clusters. For example, a cluster of influenza A infection occurred in a chronic male ward of a psychiatric hospital in Taipei in 2007. Among 87 chronic psychiatric patients, 32 patients (36.8%) and 1 nurse in the ward had upper respiratory tract infectious symptoms, and the specimens of 25 cases cultivated influenza A virus. Furthermore, in five patients (15.6%) the disease was complicated by pneumonia [21]. During the investigation period of this study, there was a statistically significant difference in the incident number of a single or pooled test with a positive case of URTIs. From 2011 to 2019, the analysis of cluster characteristics of URTIs showed that single influenza A virus was the cause of most positive events every year, which is similar to foreign literature studies [22,23]. In addition, the routine testing of the total number of clusters of URTIs from 2011 to 2019 also determined that single influenza A virus caused the most infections (64.9%, 937/1444). The second highest number of incidents was caused by single influenza B virus (22.5%, 325/1444). Although the results showed that single influenza B virus was responsible for the highest number of cluster events in 2011 and 2014, during the entire investigation period, the main pathogen causing URTIs in Taiwan was still single influenza A virus. The study indicated that the pathogens causing URTI clusters in Taiwan may change from influenza A virus to B virus and then back to A virus, depending on the time period. Outbreaks change over time, increasing the burden on public health and anti-epidemic personnel while also causing physicians to distort clinical differential diagnosis.

Doi et al. reported that, in Japan, most of the clusters of URTIs caused by non-influenza respiratory viruses in 2014 were due to respiratory syncytial virus [24]. Our study showed that most of the clusters in Taiwan in 2014 were also due to the respiratory syncytial virus. The inference of these findings may be that both Taiwan and Japan are developed countries, and their national conditions, lifestyles, healthcare in medical institutions, and quality of community and family hygiene are similar; thus, the number of incidents of URTIs caused by non-influenza respiratory viruses is roughly the same. In addition, this study revealed that both the clusters of URTIs over the past 9 years, and the number of clusters caused by non-influenza respiratory viruses, vary from year to year. The main viruses were adenoviruses from 2011 to 2012, respiratory syncytial virus from 2013 to 2016, adenoviruses from 2017 to 2018, and respiratory syncytial virus in 2019. This trend of ebb and flow may explain the interaction between humans in a community environment and non-influenza respiratory viruses in Taiwan, and it also makes the pathogenic role of the URTI cluster more diverse and difficult to identify. This is a serious issue for epidemic prevention. Taiwan’s official health department must further evaluate or develop public health intervention plans to prevent or effectively slow down influenza clusters in the future.

In Taiwan, clusters of URTIs occur in crowded, confined spaces. During the investigation period of this study, it was shown that there was a statistically significant difference in the number of incidents of URTI clustering in different types of locations. In 2011, 2013–2014, and 2017–2019, schools were the location of the majority of URTI clusters. In 2012, 2015, and 2016, densely populated institutions were the main locations. The results of this study not only showed that schools and densely populated institutions were the most common places for URTIs to cluster, they also clarified that the annual variation in cluster locations makes effective prevention and control difficult. This increases the burden on society and depletes medical resources. It is necessary to monitor the changing trends of locations of clusters over a long period and conduct follow-up research. Schools accounted for 41.6% of all the URTI clusters reported to TNIDSS. A higher percentage of URTI clusters in schools was also reported in China [25]. However, this differs from the United States, where acute and long-term healthcare facilities (e.g., nursing homes) were reported as the most common setting for URTI outbreaks [26]. This difference in primary outbreak setting may reflect the greater number and size of long-term care facilities in the United States and Europe compared to Taiwan. With a mean of 35–50 students per class, schools are likely more densely populated in Taiwan than in other countries. Close contact between students can facilitate person-to-person transmission, particularly among young children with lower levels of hand hygiene. The high numbers of URTI clusters at schools resulting from person-to-person transmission underscores the importance of isolating ill persons, improving hand hygiene, and conducting proper environmental disinfection.

Complete sequences were obtained for four influenza A(H1N1) virus samples isolated from cases that occurred during the 2017 epidemic season in Kerala, South India. Sequence analysis showed mutations that differentiate this strain from the reference strain A/California/07/2009 virus [27]. The results of the above-mentioned literature research are similar to this study’s survey of the influenza H1N1 virus epidemic in Taiwan. Recently, another study reported that in coastal Kenya, out of 186 (90 inpatient and 96 outpatient) influenza A virus-positive specimens processed, 101 A(H3N2) virus whole genomes were obtained. Among the viruses identified in inpatient specimens from 2009 to 2015, the divergence of circulating A (H3N2) viruses from the vaccine strains A/Perth/16/2009, A/Texas/50/2012, and A/Switzerland/9715293/2013 formed six genetic clades (A/Victoria/208/2009-like, 3B, 3C, 3C.2a, 4, and 7). Among viruses identified in outpatient specimens from 2015 to 2017, there was divergence of circulating A(H3N2) viruses from vaccine strain A/Hong Kong/4801/2014 [28]. The results of the above study were also similar to the current survey of the influenza H3N2 virus epidemic in Taiwan in this study. In China, a study reported the isolation of two human influenza virus strains—A/Hebei/F076/2018(H1N1) and B/Hebei/16275B/2018—from patients with severe influenza in Hebei, China, during the flu season in January 2018. B/Hebei/16275B/2018 belongs to the Victoria lineage and is closely related to the World Health Organization reference strain B/Brisbane/60/2008 [29]. After categorization of isolated virus strains by gene sequencing in this study, it was found that of seasonal influenza A viruses, H1N1 subtype viruses were predominantly A/California/07/2009, A/Michigan/45/2015, and A/Brisbane/02/2018, and H3N2 subtypes were predominantly A/Hong Kong/4801/2014, A/Singapore/INFIMH-16–0019/2016, and A/Switzerland/8060/2017, during 2017–2019. Of the influenza B viruses, B/Brisbane/60/2008 (B/Vic) was the dominant type, and some were B/Massachusetts/02/2012 (B/Yam) and B/PHUKET/3073/2013 (B/Yam). The results of the above-mentioned study in China are also similar to the current investigation of the influenza B virus epidemic in Taiwan in this study. From the above results, it can be seen that the Earth is essentially one continuum; influenza outbreaks have no borders; and recent frequent international exchanges, global climate change, and changes in living environments have caused the emergence of various novel viruses. Infectious diseases, especially respiratory infectious diseases, are spreading rapidly all over the world, and their occurrence is becoming more frequent and serious. Therefore, a multi-sectorial approach is required, including international epidemic surveillance, government agencies’ efficient epidemic prevention policies, the prevention of overseas immigration of epidemics, the expansion of medical services in local medical institutions, and community awareness, all working together to ensure effective epidemic control measures.

In recent years, due to the increase in public healthcare awareness, large-scale URTI cluster incidents have often attracted public attention. For example, in July 2019, an outbreak of influenza-like illness or a cluster of URTIs occurred in a college in Kaohsiung City, Taiwan. At least 100 people developed symptoms of URTIs, such as fever and cough, and 109 people were diagnosed with influenza-like illness [30]. In the face of large-scale or focused cluster incidents, the rapid diagnosis of the source of infection can help with epidemic control. However, from 2011 to 2019, Taiwan reported 1864 clusters of URTI, of which 341 were not detectable in routine tests. This showed that the current routine test for URTIs defined by the Taiwan CDC can detect about 80% of the pathogens of URTIs. In addition, non-routine testing requires a higher cost of inspection, and it is recommended that it be applied to emergencies or large clusters.

Because the influenza virus changes very rapidly, in addition to mastering the distribution of the number of cases, the monitoring of influenza also requires efficient laboratory testing to identify the type, variability, transmission, and pathogenicity of the virus. In addition, complete influenza virus surveillance will not only help to master the source and route of disease transmission but also help to diagnose the disease and confirm the appropriateness of antiviral drugs, so as to improve the medical quality, control the epidemic and transmission of influenza, and reduce the social cost of medical treatment. Taking this study as an example, based on the observation and recording of the relevant data on URTI clusters in the “statistical query and monitoring network of infectious diseases” published on the internet, this study uses empirical public health science methods to explore the epidemiological characteristics, differences, or trends in URTI clusters over the years. It applies the medical evidence available to public health services in order to prevent or slow down outbreaks of clusters of URTIs, evaluate exposure to hazards, or change the lifestyles of susceptible people through health education, so as to improve people’s health, which is necessary.

This study has some main limitations. Firstly, the “statistical data of infectious diseases” published by the Taiwan CDC on the network platform only provides basic epidemiological data about URTI cluster events; there are no clinical data. Therefore, this study cannot compare the differences or trends in patients’ clinical data or symptoms. However, this study provides authentic positive confirmed cases announced by the Taiwan CDC. Second, this study only monitors the influenza virus by collecting samples from designated doctors, and from outpatient, emergency, and inpatient patients suspected to be infected with influenza virus, in medical centers where national and contract laboratories are located. There is no information on the genotypes of influenza viruses or other respiratory virus strains in patients with upper respiratory tract clusters. In addition, the genetic relationship between domestic and foreign virus strains cannot not be elucidated. Third, the definition of influenza cases is based on information on the official website of the Taiwan CDC, which does not specify what temperature is used to define fever (i.e., >38 °C). Therefore, we are not sure of the definitive clinical definition of fever. Fourth, the definition of influenza cases based on the information on the official website of the Taiwan CDC does not specify a time-variant, which is necessary in cases where a different symptom appears in the same child within 14 days from the first symptom after an initial recovery. Therefore, we cannot be sure if these are part of the same case or a new case. These limitations are not of significant importance in relation to this study and the interpretation of the results, and said limitations may not affect the validity of our results. Our findings strongly recommend that government institutions collect and publicly announce detailed pathological causes and clinical criteria of UTRI clusters and allow researchers to use more complete data to analyze them in-depth. One advantage of this study is that the existing network platform of Taiwan’s health department keeps data regarding URTI clustering for many years, so that public health (clinical) researchers or institutions can use these data to enhance scientific research on infectious disease epidemiology in Taiwan.

These results may be worthy of attention from the government health departments for use in decision-making or as a reference for clinical or epidemiological expert research. Our results suggest that government departments should continue to support new laboratory techniques and diagnostic standards to increase knowledge on epidemiological characteristics and data. This will help with tracking URTI cluster incidence and discussing their epidemic patterns, trends, and risk factors in the future.

## Figures and Tables

**Table 1:** The characteristics of influenza virus and other pathogens by analyzed URTI cluster data from 2011 to 2019, Taiwan.

Pathogens	Year									
	2011 *n* = 76	2012 *n* = 72	2013 *n* = 118	2014 *n* = 154	2015 *n* = 178	2016 *n* = 166	2017 *n* = 358	2018 *n* = 255	2019 *n* = 487	2011–2019 *n* = 1864
Single influenza A virus										
Influenza A (H1N1) virus	14	5	30	17	16	43	5	16	75	221
Influenza A (H3N2) virus	1	31	34	21	95	30	200	76	58	546
Influenza A virus—RIDTs analysis	0	0	0	0	4	2	34	23	107	170
Single influenza B virus										
Influenza B virus	26	9	1	48	26	13	32	41	72	268
Influenza B virus—RIDTs analysis	0	0	0	0	0	0	2	10	45	57
Mixed Influenza virus										
Influenza A (H1N1) virus + Influenza A (H3N2) virus	0	1	0	1	0	1	0	1	1	5
Influenza A (H1N1) virus + Influenza B virus	1	0	0	0	0	1	1	0	0	3
Influenza A (H3N2) virus + Influenza B virus	1	0	0	2	1	0	6	1	1	12
Influenza A virus—RIDTs analysis + Influenza B virus—RIDTs analysis	0	0	0	0	0	0	2	0	11	13
Other pathogens	14	5	15	13	8	20	28	31	15	149
Routine test—pathogens not detected	15	14	27	49	25	56	38	52	65	341
Sample not collected	4	7	11	3	3	0	10	4	37	79

URTI: upper respiratory tract infection. Descriptive data are shown as count numbers.

**Table 2:** The risk factors of influenza virus and other pathogens from analyzed URTI cluster data from 2011 to 2019, Taiwan.

Pathogens	Year									*p*
	2011 *n* = 57	2012 *n* = 51	2013 *n* = 80	2014 *n* = 102	2015 *n* = 150	2016 *n* = 110	2017 *n* = 310	2018 *n* = 199	2019 *n* = 385	
Single influenza A virus	15	36	64	38	115	75	239	115	240	<0.001
Single influenza B virus	26	9	1	48	26	13	34	51	117	
Mixed influenza virus	2	1	0	3	1	2	9	2	13	
^a^ Other pathogens	14	5	15	13	8	20	28	31	15	

^a^ Other pathogens included respiratory syncytial virus, adenovirus, enterovirus, rhinovirus, human metapneumovirus, parainfluenza virus, and mycoplasma; URTI: upper respiratory tract infection. Categorical variables are shown as counts and compared using the chi-squared test.

**Table 3:** The risk factors of non-influenza respiratory virus from analyzed URTI cluster data from 2011 to 2019, Taiwan.

Pathogens	Year									*p*
	2011 *n* = 14	2012 *n* = 5	2013 *n* = 15	2014 *n* = 13	2015 *n* = 8	2016 *n* = 20	2017 *n* = 28	2018 * n = 22	2019 *n* = 15	
Respiratory Syncytial Virus	4	2	10	6	5	11	10	8	8	0.01
Adenovirus	6	2	3	5	1	5	15	11	6	
Enterovirus	2	0	2	0	2	2	3	3	1	
Rhinovirus	0	0	0	2	0	0	0	-	0	
Adenovirus + Respiratory Syncytial Virus	1	0	0	0	0	0	0	-	0	
Enterovirus + Respiratory Syncytial Virus	0	1	0	0	0	0	0	-	0	
Human Metapneumovirus	0	0	0	0	0	1	0	-	0	
Adenovirus + Human Metapneumovirus	0	0	0	0	0	1	0	-	0	
Parainfluenza Virus + Mycoplasma	1	0	0	0	0	0	0	-	0	

URTI: upper respiratory tract infection; *: 2018 year includes 22 single infections and 9 mixed infections (data not shown). -: not applicable. Categorical variables are shown as counts and compared using the chi-squared test.

**Table 4:** The risk factors of institutions from analyzed URTI cluster data from 2011 to 2019, Taiwan.

InstitutionCategories	Year									*p*
	2011 *n* = 76	2012 *n* = 72	2013 *n* = 118	2014 *n* = 154	2015 *n* = 178	2016 *n* = 166	2017 *n* = 358	2018 *n* = 255	2019 *n* = 487	
Schools	38	11	64	89	62	38	165	127	339	<0.001
Populous institutions	24	37	37	37	70	64	135	86	91	
Hospitals	8	18	12	25	38	61	45	38	44	
Military camps	4	3	2	2	6	3	8	2	9	
Others ^a^	2	3	3	1	2	0	5	2	4	

^a^ Others included family, company, and tour groups; URTI: upper respiratory tract infection. Categorical variables are shown as counts and compared using the chi-squared test.

**Table 5:** The categorization of isolated influenza virus strains by gene sequencing in Taiwan during 2011 and 2019.

Variables	Year								
	2011	2012	2013	2014	2015	2016	2017	2018	2019
Influenza A virus									
HIN1									
A/California/07/2009	√	√	√	√	√	√	√		
A/Michigan/45/2015						√	√	√	
A/Brisbane/02/2018									√
H3N2									
A/Perth/16/2009	√								
A/Victoria/361/2011		√	√						
A/Texas/50/2012				√		√			
A/Switzerland/9715293/2013				√		√			
A/Hong Kong/4801/2014					√		√	√	√
A/Switzerland/9715293/2013					√				
A/Singapore/INFIMH-16–0019/2016							√	√	√
A/Switzerland/8060/2017								√	√
Influenza B virus									
B/Brisbane/60/2008 (B/Vic)	√	√	√	√	√	√	√	√	
B/Florida/4/2006 (B/Yam)	√								
B/Malaysia/2506/2004 (B/Vic)	√								
B/Wisconsin/01/2010 (B/Yam)		√	√	√					
B/Massachusetts/02/2012 (B/Yam)			√	√	√	√	√	√	
B/PHUKET/3073/2013 (B/Yam)				√	√	√	√	√	√
B/Colorado/06/2017 (B/Vic)								√	
B/Colorado/06/2017 (B/Vic)									√
B/Washington/02/2019 (B/Vic)									√

Ticks are the main epidemic influenza strains each year.

## Data Availability

Not applicable.
